# Enhancing brief intervention with motivational interviewing in primary care settings

**DOI:** 10.1186/1940-0640-7-S1-A12

**Published:** 2012-10-09

**Authors:** Christopher Dunn, Sarah G Trusz, Kristin Bumgardner, Peter Roy-Byrne

**Affiliations:** 1 Harborview Medical Center, University of Washington, Seattle, WA, USA; 2 Department of Psychiatry and Behavioral Science, University of Washington School of Medicine, Seattle, WA, USA

## 

Training in screening brief intervention, and referral to treatment (SBIRT) for substance abuse is being widely disseminated and implemented in a variety of health-care settings. Use of motivational interviewing (MI) techniques is thought to enhance BI effectiveness. The science of BI-MI training has not yet established optimal training doses for interventionist trainees to reach beginning competence. This study evaluated two training packages for teaching BI-MI, an eight-week comprehensive and a four-week accelerated training in primary care medical settings. Interventionist trainees were medical social workers (n = 22) in primary care clinics serving safety-net patients with drug abuse. Trained coders evaluated post-training BI and MI performance during standardized patient role-play interviews using a checklist of BI clinical tasks and the Motivational Interviewing Treatment Integrity (MITI) 3.0 coding system. Both training models yielded similar end-point MI skill levels. The proportion of learners who reached beginning proficiency on MI skills by the end of training was comparable to that reported in similar MI training studies (between 25% and 65%). Results suggest that some practitioners working in busy medical settings can learn BI and reach beginning proficiency in MI in as little as one month. Adherence to BI content and MITI outcomes with patients in primary care settings will also be presented.

